# Revisiting the application of Immobilized Artificial Membrane (IAM) chromatography to estimate *in vivo* distribution properties of drug discovery compounds based on the model of marketed drugs

**DOI:** 10.5599/admet.757

**Published:** 2020-01-31

**Authors:** Klara Valko, Silvia Rava, Shenaz Bunally, Scott Anderson

**Affiliations:** 1 Bio-Mimetic Chromatography Ltd. Business and Technology Centre, Bessemer Drive, Stevenage, SG1 2DX, United Kingdom; 2 University of Pavia, Italy, Erasmus internship at Bio-Mimetic Chromatography Ltd. Business and Technology Centre, Bessemer Drive, Stevenage, SG1 2DX, United Kingdom; 3 Physicochemical Group, GSK, Gunnels Wood Road, Stevenage, SG1 2NY United Kingdom; 4 Regis Technologies Inc. 8210 Austin Avenue Morton Grove, IL US 60053

**Keywords:** Phospholipid binding, HPLC, volume of distribution, tissue binding, drug efficiency

## Abstract

Immobilized Artificial Membrane (IAM) chromatography columns have been used to model the in vivo distribution of drug discovery compounds. Regis Technologies Inc., the manufacturer, had to replace the silica support and consequently introduced a new IAM.PC.DD2 column that shows slightly different selectivity towards acidic and basic compounds. The application of the new IAM.PC.DD2 columns has been evaluated and the in vivo distribution models have been compared with the previous batches of columns. It was found that due to the improved endcapping of the silica, some of the positively charged drug molecules showed shorter retention than previously published. Therefore, the column system suitability data have been updated. However, these differences do not significantly affect the previously published models for the volume of distribution, brain tissue binding and drug efficiency. Therefore, the published models can be used with the new IAM.PC.DD2 columns.

## Introduction

Pidgeon and Venkataran [1] patented a method for immobilizing phospholipids on HPLC-grade silica stationary phases. The immobilized phospholipids mimic the lipid environment of a fluid cell membrane on a solid matrix. The retention factors (*k*) of compounds obtained on the IAM stationary phase are proportional to their affinity (partition coefficient) (*K*) to phospholipids according to the equation (1):


(1)

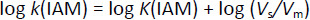



where *k* is the retention factor obtained from the retention time (*t*_R_) and the dead time (*t*_0_) according to equation (2)


(2)

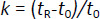



and *V*_s_*/V*_m_ is the volume ratio of the stationary and mobile phases, respectively. Many drug molecules bind strongly to phospholipids as it is a pre-requisite for membrane permeability. To reduce the retention times various concentrations of acetonitrile can be used in the mobile phase. There is a linear relationship between the log *k* values and the percentage of acetonitrile in the mobile phase that allows the extrapolation of the retention factor to the zero percentage of acetonitrile, i.e. to the pure aqueous buffer (log *k_0_*) as shown by equation 3.


(3)

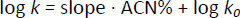



Equation 3 enables the calculation of the acetonitrile concentration that is necessary to achieve  log *k* = 0, when the retention time is exactly double that of the dead time, which means an equal distribution of the compound in the mobile and the IAM stationary phase [2]. This acetonitrile concentration expressed in volume percentage is called the Chromatographic Hydrophobicity Index (CHI (IAM)) [3]. It has also been shown that the CHI (IAM) values have linear relationships with the gradient retention times of the compounds obtained on the IAM stationary phases. By using an acetonitrile gradient of 0% to a maximum of 85% the obtained gradient retention times can be calibrated from the CHI (IAM) values by using a set of standards, eliminating the need to carry out isocratic measurements. The so obtained CHI (IAM) values show a good correlation to the log *k_0_* (IAM) values, that are the extrapolated logarithmic retention factors to 0% acetonitrile concentration in the isocratic mode. The chromatographic determination of phospholipid-binding is based on measuring gradient retention times. It is independent of the amount of compound injected onto the column, so there is no need for quantitative analysis. The time measurement is very reproducible, and the calibrated (relative) gradient retention data are suitable for inter-laboratory comparison and application in published models of *in vivo* drug distribution. The data so obtained can be compiled in databases and is suitable for use in establishing quantitative structure- retention relationships, thus enabling chemists to design compounds with the appropriate IAM binding.

The application of IAM chromatography as an aid in drug discovery has been reviewed extensively in recent years [4-8]. The molecular factors that influence IAM retention have also been discussed [9, 10]. It has been found that IAM columns retain strongly positively charged compounds as the IAM columns are negatively charged on the surface similar to cell membranes [11-13]. Several applications of IAM chromatography have also published [14] and this is summarized below.

Intestinal absorption and drug distribution depend on a compound’s partitioning into phospholipids. Compounds have to possess a certain degree of affinity to membranes in order to permeate through the biological phospholipid bilayers. Therefore, IAM chromatography can provide an insight into the potential intestinal absorption of compounds [15]. As tissues represent a more non-polar lipid environment relative to the plasma, the human volume of distribution can be modelled by the binding differences between IAM and human serum albumin (HSA) [13, 16], as shown by equation (4):


(4)

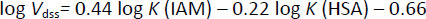



The sum of the albumin and phospholipid-binding inversely correlates to the unbound fraction of the compounds in tissues according to equation (5) [17].


(5)

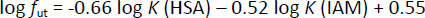



The *in vivo* drug efficiency can be modelled by the sum of the IAM and HSA binding of compounds [18] according to equation 6. The *in vitro* DRUG_eff_ shows an excellent correlation with the *in vivo* DRUG_eff._ Drug efficiency and potency enables early dose estimation as drug efficiency approximates to the proportion of free drug concentration and dose [19].


(6)

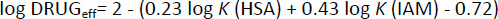



It has been found that basic compounds that have CHI (IAM) value greater than 50 have phospholipidosis potential [20] and also show promiscuous binding, interacting with several targets, causing potential toxicity and side effects [21].

At present Regis Technologies Inc. (Morton Grove, IL, USA) is the only manufacturer of the Immobilized Artificial Membrane (IAM) stationary phase. The stationary phase contains only a monolayer of the phosphatidylcholine (PC) with the aliphatic part chemically bonded to the silica while the choline polar head group faces towards the mobile phase. Initially, “Type A” silica was used in the bonding procedure. The PC molecule has only one alkyl chain available with the second alkyl chain directly bonded to the silica surface. In this way, a denser bonding could be achieved which was closer to the natural density of PC molecules on the cell surface. However, in 2018 the company had to change the base silica material to a newer “Type B” as the old supply was no longer available. “Type B” silica has been chosen as the closest in properties to “Type A” regarding the specific surface area, pore size and particle size. When testing the retention selectivity of the new IAM.PC.DD2 columns which were otherwise manufactured in the same way, differences were observed towards acidic and basic molecules. This was somewhat surprising as the new silica support had identical physical specifications to the previously used silica (10 μm particle size, 300 Å pore size and nearly identical surface area). However, given the newer silica is of a much higher purity with only single ppm metal content, these observations are understandable as the new silica provides a much more inert surface and likely contributes to less non-specific binding interactions.

The difference in selectivity can also be attributed to the different arrangements of the positive and negative charges of the choline headgroup on the stationary phase surface, again somewhat affected by the higher purity silica during the bonding process. The new batches of columns did not pass the original system suitability test criteria. Hence the aim was to establish new CHI (IAM) values for the system suitability test compounds that could be reproduced on the new batches of IAM columns. In this paper, the investigation of the selectivity difference is reported using over 70 marketed drug molecules and the *in vivo* distribution models are compared for the old and the new batches of IAM.PC.DD2 stationary phases. The estimated volume of distribution data was compared with the data from the previously published models. The differences of the models for the estimated brain tissue binding and drug efficiency values of the known drug molecules are investigated and compared with the published data obtained using the previous batches of IAM.PC.DD2 columns.

## Experimental

All high-performance liquid chromatography measurements were performed on an Agilent 1100 Series HPLC instrument equipped with an ultraviolet diode array detector (DAD).

### Measurements of membrane binding using immobilized artificial membrane (IAM) chromatography

The IAM binding was measured using IAM.PC.DD2 HPLC columns (Regis Technologies, Inc., IL, USA) with dimensions of 100 x 4.6 mm, particle size 10 μm with 300 Å pore size.

Two batches of IAM.PC.DD2 columns were studied (R20511-014-3 and P20511-014-3) that had been prepared using the new batches of silica support. The selectivity and the models obtained were compared with the published average IAM data obtained on the columns manufactured between 2004 and 2012 [14].

Mobile phase A was 50 mM ammonium acetate adjusted to pH 7.4 and mobile phase B was 100% acetonitrile. The mobile phase flow rate was 1.5 mL/min and the run time was 6 min. The acetonitrile gradient was applied from 0 to 85% from 0 to 4.75 min and kept at 85% until 5.15 min. From 5.15 to 5.25 min the acetonitrile concentration dropped back to 0%.

The retention times were standardized using the IAM calibration mixture, containing octanophenone, heptanophenone, hexanophenone, valerophenone, butyrophenone, propiophenone, acetophenone, acetanilide and paracetamol. The retention times of the compounds were plotted against the isocratically determined and predefined CHI (IAM) values [4] listed in Table 1. The slope and intercept of the obtained straight line was used to calculate the CHI IAM values of new compounds. The CHI IAM values were converted to the octanol/water lipophilicity scale and expressed as log *K* (IAM) values using equation (7).


(7)

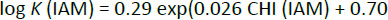



**Figure 1.**
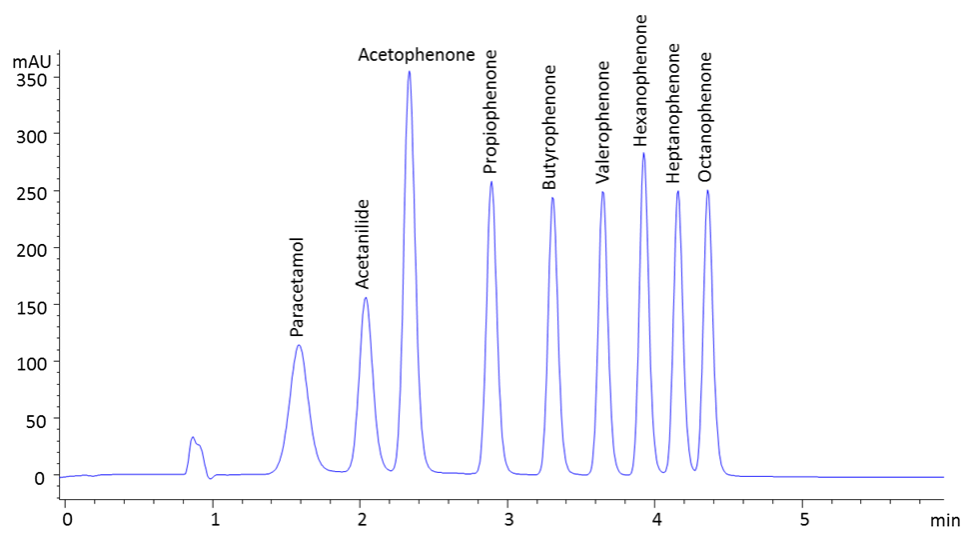
A typical chromatogram obtained on IAM.PC.DD2 100 x 4.6 mm HPLC column. Flow rate: 1.5 mL/min; Mobile phase A: 50 mM ammonium acetate adjusted to pH 7.4, mobile phaseB: acetonitrile. Run time 6 min; Gradient: 0 to 4.75 min 0 to 85% B, 4.75 to 5.15 min 85% B, 5.15 to 5.25 min 0% B; Detection wavelength: 250 nm

The so-obtained log *K*_IAM_ values express the membrane partition comparable to the octanol/water lipophilicity as described in reference [22]. A typical chromatogram is shown in Figure 1.

The natural state of the phosphatidylcholine headgroup on the IAM stationary phase provides the column selectivity that was tested using the system suitability test compounds containing acidic, basic and neutral compounds.

### Protein binding measurements using biomimetic protein stationary phases

The interaction of the compounds listed in Table 2 with human serum albumin (HSA) has been measured using commercially available chemically bonded HSA columns (Chiralpak-HSA from HiChrom, UK) with the dimensions of 50 x 3 mm and a particle size of 5 μM.

Mobile phase A was 50 mM ammonium acetate adjusted to pH 7.4 and mobile phase B was 2-propanol. The flow rate was 1.5 mL/min and the run time was 6 min. The 2-propanol gradient was applied from 0 to 35% from 0 to 3.00 min and kept at 35% until 4.00 min. From 4.00 to 4.25 min the 2-propanol concentration was dropped back to 0%. Although column manufacturers suggest using pH 7 for chiral separations using the HSA column, in this case, the pH 7.4 mobile phase is used to mimic the plasma pH for the plasma protein binding measurements.

### Compounds studied

In this study, 72 commercial drugs from diverse therapeutic areas have been analysed. 50 μl of 10 mM DMSO solutions of the drugs were diluted down to 150 μl and 10 μl of the diluted solution was injected onto the HPLC columns. The clinical volume of distribution data for all of the drugs is available in the literature [13].

Table 2 shows the marketed drug molecules and their clinical volume of distribution data obtained from reference [13] and the previously measured CHI (IAM) data [14] using an IAM column manufactured by Regis Technologies between 2004 and 2012.

### Data analysis

The statistical analysis was performed using JMP 13 (SAS Institute, USA).

## Results and Discussion

The selectivity and reproducibility of IAM.PC.DD2 columns have been extensively studied. First, the CHI (IAM) values of the system suitability test compounds (see Table 2) were scrutinized. The CHI (IAM) values of the test compounds were then compared with the CHI (IAM) values obtained on the new batches of IAM.PC.DD2 columns. It was found, as shown in Table 3, that carbamazepine, warfarin, indomethacin, nicardipine, ketoprofen, haloperidol and budesonide had very similar CHI (IAM) values. However, imipramine and chlorpromazine showed greater than the 5 CHI (IAM) unit deviations which would be within the experimental error and therefore they did not pass the system suitability criteria. These compounds are strong bases and positively charged at pH 7.4. Most probably the new IAM.PC.DD2 columns have less negative charge on the surface and therefore reduce the retention times of positively charged compounds. It is also possible that more of the free silanol groups were end-capped which would contribute to a shorter retention of basic compounds. Therefore, the system suitability criteria were changed for basic compounds. The “new” CHI (IAM) values are listed in Table 3.

The *in vivo* distribution models were re-examined using the data from the new IAM.PC.DD2 columns. Because of the selectivity differences, it was essential to investigate the CHI (IAM) values for a larger set of compounds and check how the previously described *in vivo* distribution models performed using the data from the new columns manufactured with the new silica material.

The 72 drug molecules from the original set of marketed drugs for which the models were developed [13] were analysed on the new IAM.PC.DD2 columns and the HSA column. Table 4 shows the CHI (IAM) values and the log *k* HSA binding data obtained from reference [13] and remeasured in this study.

**Figure 2.**
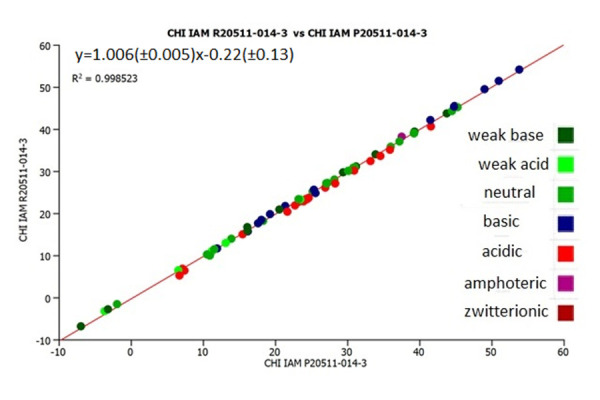
The plot of the experimental CHI (IAM) values on IAM.PC.DD2 columns P20511-014-3 and R20511-014-3

Figure 2 shows the plot of the experimental CHI (IAM) values on IAM.PC.DD2 columns P20511-014-3 and R2055-014-3. The CHI (IAM) values obtained on the columns are very similar indicating good batch to batch reproducibility of the new columns.

**Figure 3.**
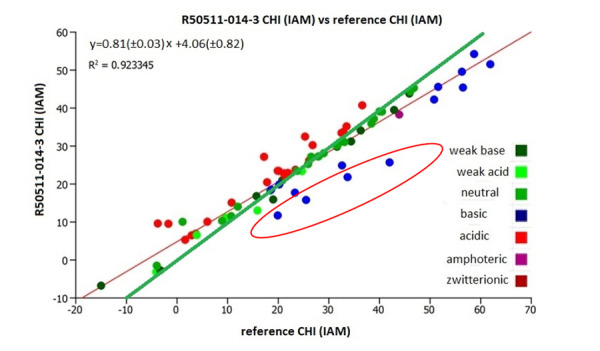
The plot of the CHI (IAM) values from the literature [13] (reference CHI (IAM)) with the CHI (IAM) values using IAM.PC.DD2 column R25011-014-3. The green line is the line of unity.

**Figure 4.**
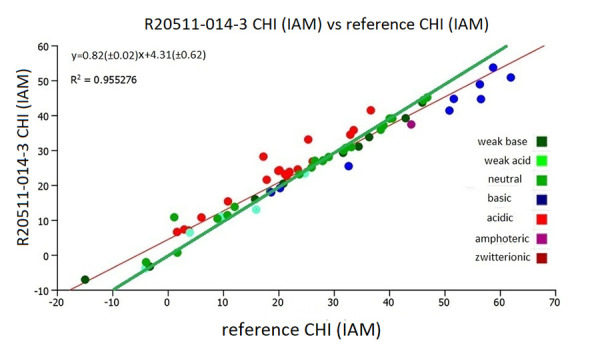
Comparison of the experimental CHI (IAM) values obtained on the IAM.PC.DD2 (R20511-014-3) column with the reference CHI (IAM) values without the 4 strong basic compounds. The green line is the line of unity

Figure 3 shows the plot of the CHI (IAM) from the literature [13], reference CHI (IAM), with the experimental CHI (IAM) values using the IAM.PC.DD2 column R2055-014-3. The CHI (IAM) values of procainamide, sulpiride, zolmitriptan and pindolol were outliers from the correlation with lower CHI (IAM) values on the new batches of IAM columns. Compounds are considered outliers when the difference between the measured and predicted value is greater than the double of the standard error of the estimate, in this case 7.2 CHI IAM units. The slope of the regression line (red) is less than one (0.81) and the regression line is different from the line of unity (green). When these 4 strong basic compounds are left out from the correlation, the correlation coefficient increases to 0.96 and the slope increases to 0.82 as shown in Figure 4.

The results shown in Figure 4 suggests that the rank order of the phospholipid binding will be very similar using the CHI IAM data obtained on the new IAM.PC.DD2 column and the old IAM.PC.DD2 column. However, some basic compounds may show significantly weaker phospholipid binding. The absolute CHI IAM values maybe lower when the new batches of IAM columns are used.

### The human clinical volume of distribution and protein binding data of the investigated marketed drugs

The volume of distribution model is based on two properties of the compounds, the IAM binding and the human serum albumin (HSA) binding. Therefore, the IAM binding data obtained using the new IAM.PC.DD2 columns were examined in the models of the human clinical volume of distribution together with the re-measured protein binding data.

**Figure 5.**
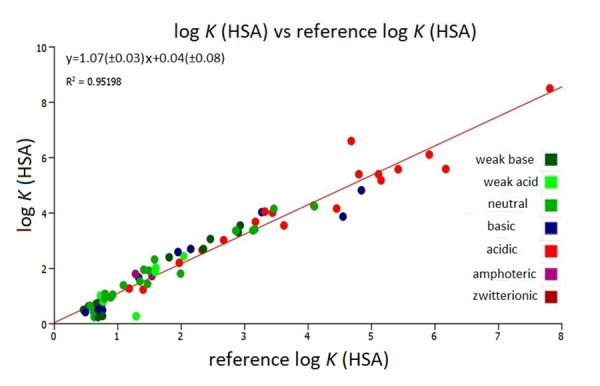
The plot of the log *K* (HSA) values from the literature [13] and the re-measured log *K* (HSA) for this study

Figure 5 shows the reproducibility of the HSA columns by plotting the newly measured log *K* (HSA) values as a function of the “reference log *K* (HSA) “ values published previously [13]. It can be seen that the slope is very close to 1 and the intercept is very close to 0. There are slightly greater discrepancies between the reference and the re-measured log *K* (HSA) values for strongly bound compounds. This shows that the specific binding sites of the human serum albumin may vary only slightly from column to column. With this excellent agreement between the log *K* (HSA) values obtained on various Chiral HSA columns, it can be assumed that they would not affect the volume of distribution model which is based on two binding properties for the compounds, namely the IAM and HSA binding.

### Estimating the volume of distribution using the old and new IAM.PC.DD2 columns

The logarithmic values of the HSA and IAM binding data have been used to estimate the human clinical volume of distribution. Using the published equation (equation 4), the estimated values are compared using the IAM data obtained on columns R20511-014-3 and P20511-014-3 and the IAM data from the literature [12].

The estimated log *V*_dss_ values have been calculated using equation 4 and the measured log *K* (HSA) and log *K* (HSA) data obtained on the new IAM.PC.DD2 columns. The estimated log *V*_dss_ values are listed in Table 5 together with the acid/base character of the compounds.

**Figure 6.**
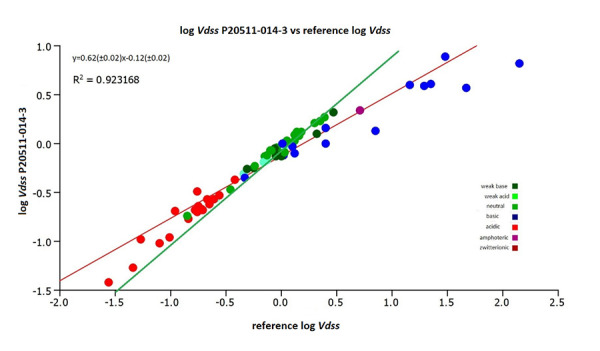
The plot of the estimated log *V*_dss_ values using the CHI (IAM) values obtained on the IAM.PC.DD2 column P20511-014-3 and from the reference [13]. The green line is the line of unity.

Figure 6 shows the plot of the estimated log *V*_dss_ values using the CHI (IAM) values obtained on the IAM.PC.DD2 column P20511-014-3 and those derived from the reference CHI (IAM) values. A good correlation was found between the estimated log *V*_dss_ values. In this study equation 4 was used with the same log *K* (HSA) values and different log *K* (IAM) values (depending on which IAM column was used).

**Figure 7.**
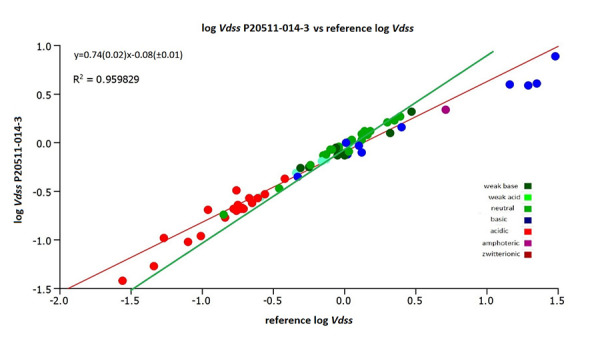
The plot of the estimated log *V*_dss_ values using the CHI (IAM) values obtained on column P20511-014-3 and from the reference values [12] without the four strong basic compounds. The green line is the line of unity.

Figure 7 shows the plot of the estimated log *V*_dss_ values using the CHI (IAM) values obtained on the IAM.PD.DD2 column P20511-014-3 and those obtained from the reference but without the 4 strong basic compounds (procainamide, sulpiride, zolmitriptan and pindolol). It can be seen that the agreement is slightly improved with an R^2^ value of 0.96, however, the slope is still less than one, while the intercept is close to zero. This means that the rank order of the volume of distribution data would be the same when using the new batches of IAM.PC.DD2 columns as it was when using the old batches of IAM columns. However, absolute values may be slightly different for the neutral, acidic and basic compounds and greater discrepancies can be expected for some strong basic compounds which are similar to those left out from the plot.

**Figure 8.**
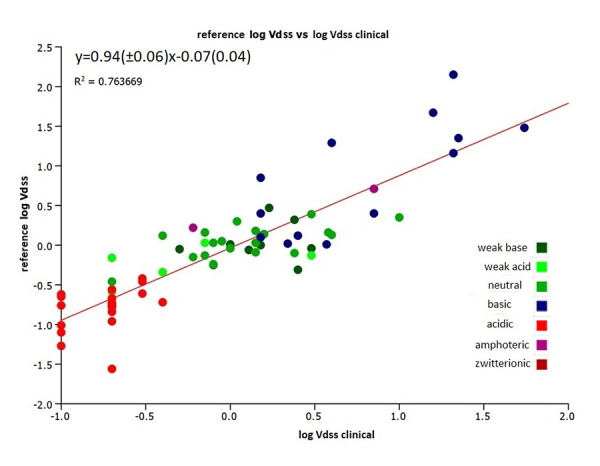
The plot of the estimated and the measured clinical volume of distribution data using the old batches of IAM columns (reference [13] reference log *V*_dss_) using equation 4.

**Figure 9.**
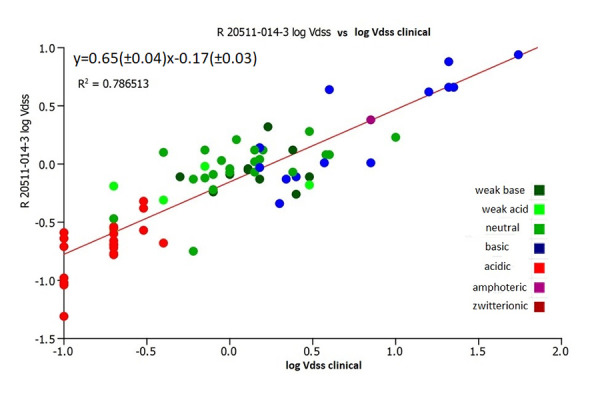
The plot of the estimated and measured clinical volumes of distribution using the new batch R20511-014-3 IAM.PC.DD2 column versus the reference log *V*_dss_ *[13]*.

It was important to investigate how the estimated volume of distribution using the CHI (IAM) data from the new batches of the IAM.PC.DD2 columns correlated with the actual clinical volume of distribution data. Figures 8 and 9 show the correlation of the estimated log *V*_dss_ values using the same coefficients as in equation 4 with the clinical log *V*_dss_ values. Figure 9 shows that the CHI (IAM) values obtained on the new batches of IAM.PC.DD2 column can be used to estimate the human clinical volume of distribution data by using equation 4. There is better agreement with the clinical data when using the log *V*_dss_ obtained on the new batch of the IAM.PC.DD2 column. However, the absolute values may be slightly different and a correction factor (slope 0.62) will be required. In a drug discovery setting for ranking and differentiating compounds using an estimated log *V*_dss_, these values can be used with confidence. However, it is important to acknowledge that certain strong basic compounds may appear to have a lower volume of distribution than expected.

It was also important to investigate how the CHI (IAM) and log *K (IAM)* values perform in other in vivo distribution models.

### Estimating brain tissue binding using the old and new batches of IAM.PC.DD2 columns

The brain tissue binding can be estimated from the sum of the albumin and the phospholipid binding as described by equation 8. The model was built on 135 drug discovery compounds from several neuroscience projects using rat brain tissue binding data obtained by equilibrium dialysis and measured HSA and IAM binding data as described in reference [12].


(8)

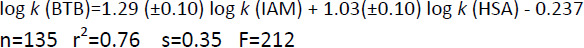



where *n* is the number of compounds, *r^2^* is the correlation coefficient, *s* is the standard error of the estimate and *F* is the Fisher-test value.

**Figure 10.**
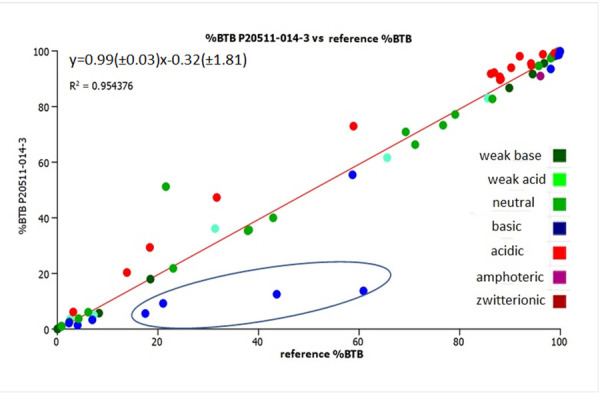
The plot of the estimated brain tissue binding (%BTB) obtained using the IAM data obtained from the reference [13] and that obtained using IAM.PC.DD2 column P20511-014-3

Figure 10 shows the comparison of the estimated brain tissue binding (%BTB) when the old (reference [13]) and the new column P20511-014-3 IAM.PC.DD2 data was used in the model.

The agreement is very good with an R^2^ value of 0.95, the slope is close to 1 and the intercept is close to zero using the CHI (IAM) values obtained on P20511-014-3 from the latest batch of IAM.PC.DD2 columns. However, the four basic compounds that showed significantly lower CHI (IAM) values are significant outliers resulting in these compounds being estimated to have lower brain tissue binding than their actual brain tissue binding.

### Estimating the unbound volume of distribution using the old and new batches of IAM.PC.DD2 columns

**Figure 11.**
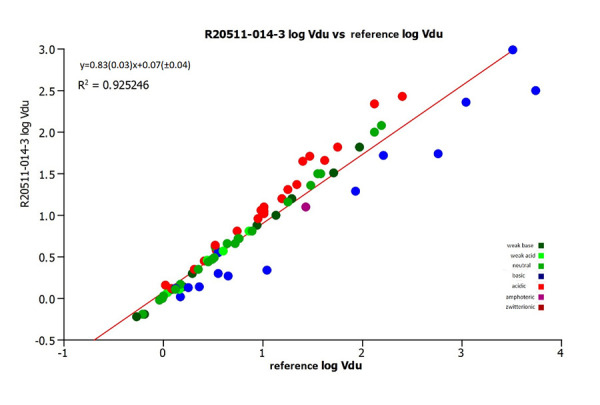
A plot of the estimated unbound volume of distribution using the IAM data from the reference (reference [12]) and on the IAM.PC.DD2 R20511-014-3 column

The agreement between the estimated unbound volume of distribution data using the reference [13] CHI (IAM) values and the CHI IAM values obtained on column R25011-014-3 are shown in Figure 11.

It can be seen that the estimated unbound volume of distribution using the CHI (IAM) values obtained on column R20511-014-3 shows good agreement with the logVdu from the reference CHI (IAM) values. It can be observed from the plot that basic compounds tend to show a lower unbound volume of distribution using the CHI (IAM) values obtained on the new column R20511-014-3.

### Estimating drug efficiency using the old and new batches of IAM.PC.DD2 columns

**Figure 12.**
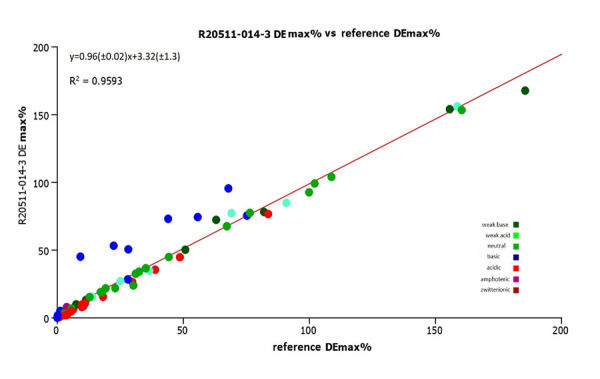
The plot of the drug efficiency values for the investigated drugs using the IAM data obtained from the reference (reference [12]) and by R20511-014-3 column

Figure 12 shows the comparison of the estimated drug efficiency values using the old and a new batch (R20511-014-3) of IAM.PC.DD2 column.

There is good agreement between the drug efficiency values obtained on the new batch of the IAM column and the literature data. However, the basic compounds showed greater drug efficiency than expected when using the new batch (R20511-014-3) of IAM.PC.DD2 column.

In conclusion, some differences between the old and the latest batches of IAM.PC.DD2 columns were observed when estimating the *in vivo* distribution of compounds. The discrepancies are more significant when the models are based on the differences between the measured IAM and HSA binding (volume of distribution). When the models are based on the sum of the two types of binding, the correlations between the estimated values (brain tissue binding, the unbound volume of distribution and drug efficiency) are much stronger. However, systematic differences could be observed for basic compounds in the estimated drug efficiency values. Polar basic (log *P* less than 2) compounds with over 6 rotatable bonds were estimated to have greater dug efficiency values than previously estimated using the old batches of IAM.PC.DD2 columns. These discrepancies are due to the weaker interaction of the new batches of IAM.PC.DD2 stationary phases with positively charged basic compounds. This could be the result of better endcapping procedures to block the free silanol groups and it could also be due to the higher purity of the silica providing less overall negatively charged silanols.

Based on these observations it was decided to investigate the potential benefit of developing new models using the data obtained on the new silica based IAM stationary phase and to compare the new models with the existing model equation (equation 4).

### Developing new distribution models using the new batches of IAM.PC.DD2 columns

The correlations between the old and the new batches of IAM retention data have been investigated. It was found that the reference CHI (IAM) data can be estimated using the CHI (IAM) data obtained on the new batches (P20511-014-3 and R20511-014-3) of IAM.PC.DD2 columns when the retention difference on C-18 columns obtained at pH 10.6 and pH 2.6 are included in the equation (equation (9)). This second variable significantly increased the explained variance of the reference CHI (IAM).


(9)

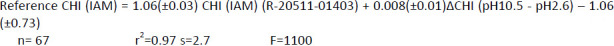



The correlation was slightly improved (from 0.92 to 0.97) between the reference CHI (IAM) values and the CHI (IAM) values obtained on the new batch of IAM.PC.DD2 columns. However, in order to be able to use equation 9, the CHI measurements on C-18 columns should be carried out at high and low pH. This requires two additional measurements for each compound. When resources are limited, the calculated molecular descriptor can be used. The following parameters have been calculated for the investigated drug molecules: log P, molecular weight, number of H-bond donor and acceptor groups, total polar surface area, flexibility and the number of rotatable bonds (NRB). It was found that the only significant variables among the calculated parameters that improved the correlation between the CHI (IAM) values obtained using the new and old batches of IAM.PC.DD2 columns were clogP and the total polar surface area (TPSA) as shown in equation 10. However, the correlation coefficient improved only slightly from 0.92 to 0.96.


(10)

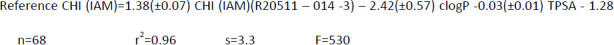



The other option was to build a new model for the estimation of the volume of distribution using the new batches of IAM.PC.DD2 columns and the clinical volume of distribution data.

The new equation for modelling clinical steady-state volume of distribution data is shown in equation 11. The mathematical-statistical parameters are very similar to the original equation (Equation 4); however, the coefficients of the IAM and HSA binding data are very different.


(11)

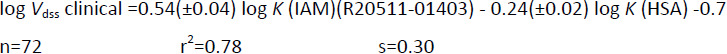



**Figure 13.**
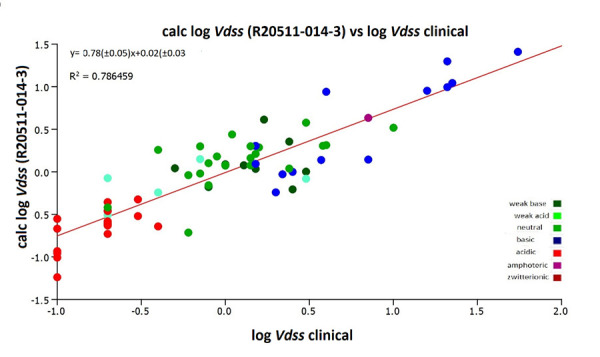
The plot of the calculated log *V*_dss_ values using the new model with the CHI (IAM) values obtained on column (R20511-014-3) as a function of the clinical log *V*_dss_ values.

The plot of the estimated and measured clinical steady-state volume of distribution data are shown in Figure 13. When comparing the coefficients of equation 4 (the published *V*_dss_ model equation) and equation 10, it can be seen that the coefficients for the HSA binding do not differ significantly (0.24 and 0.22 with an error of 0.02). However, the coefficients of log *K* (IAM) are different at the 95% confidence interval and agree only within a 90% confidence interval: 0.54 ± 0.04 and 0.44 ± 0.02.

## Conclusions

The use of a new silica support provided a slightly different selectivity for the new IAM.PC.DD2 columns regardless of the bonding procedure used. Therefore, new CHI (IAM) values have been proposed for the system suitability test compounds. New IAM.PC.DD2 columns should be evaluated using the new CHI (IAM) values (Table 4.). The reproducibility of the new batches of IAM.PC.DD2 columns are very good and are within 2 CHI (IAM) units. The effect of the selectivity change has been investigated on previously developed *in vivo* models for drug distribution that are based on CHI (IAM) values. A set of 72 marketed drugs with clinical distribution data (volume of distribution, unbound volume of distribution and drug efficiency) have been analyzed on the new batches of IAM columns. The CHI (IAM) values were compared with the values obtained from the previous batches of IAM columns and it was observed that small polar basic compounds showed significantly shorter retention times on the new IAM.PC.DD2 columns. The correlation coefficient improved significantly when the data from these compounds are removed from the analysis. The possible reason for these discrepancies may be due to the better endcapping procedure on the new IAM columns which is blocking the silanol effects that increase retention of basic compounds. It is also likely that the newer “Type B” silica is higher purity silica with a more inert surface and creates fewer negatively charged silanols decreasing the amount of non-specific binding. It was found that using the new CHI (IAM) data in the previously published model equations, a good correlation was obtained with the original values. However, the absolute values were different but the rank order for the in vivo distribution values remain the same using the old and the new columns. New models have also been developed for estimating the clinical volume of distribution of compounds using the data from the new IAM columns. The mathematical statistical characteristics of the new models are similar, but the coefficients of the CHI (IAM) and log *K* (HSA) parameters are slightly different. Since the correlation between the estimated and clinical volume of distribution data was the same or slightly better with the data obtained from the new batches of IAM columns, it is expected that the in vivo estimates can be as accurate with the data obtained from the new batches of IAM columns as with the older batches of IAM columns. The newly derived equation (eqation 11) for the estimation of the volume of distribution is suggested when someone begins to use biomimetic chromatographic measurements with the new columns. However, reference can still be made to the existing data previously published using the older columns.

## Figures and Tables

**Table 1.** The constant CHI (IAM) and log *K* (IAM) values for the calibration set of compounds with typical retention times.

Compound	CHI (IAM)	log *K* (IAM)	*t*_R_ 7.4 (min)
Octanophenone	49.4	4.97	4.37
Heptanophenone	45.7	4.30	4.17
Hexanophenone	41.8	3.71	3.93
Valerophenone	37.3	3.16	3.66
Butyrophenone	32.0	2.62	3.32
Propiophenone	25.9	2.15	2.90
Acetophenone	17.2	1.67	2.34
Acetanilide	11.5	1.45	2.04
Paracetamol	2.90	1.20	1.58

The retention times were standardized using the calibration set of compounds described previously [23].

**Table 2.** The name of the marketed drugs, their CAS number, their previously published CHI (IAM) data and the logarithm of their clinical volume of distribution.

Compound	CAS NO	Acid/base character	CHI (IAM) [14]	log *V_dss_* [13]
Acecainide	32795-44-1	basic	23.3	0.10
Acetanilide	103-84-4	neutral	10.7	-0.13
Acetazolamide	59-66-5	neutral	1.7	-0.46
Alclofenac	22131-79-9	acidic	17.8	-1.10
Amoxapine	14028-44-5	basic	56.5	1.67
Bamethan	3703-79-5	basic	18.6	0.01
Betamethasone	378-44-9	neutral	31.7	0.18
Carbamazepine	298-46-4	neutral	26.5	-0.09
Chlorpromazine	50-53-3	basic	61.9	2.15
Cinoxacin	28657-80-9	acidic	1.6	-0.42
Colchicine	64-86-8	neutral	23.7	0.03
Cytarabine	147-94-4	weak base	-15.0	-0.31
Diazoxide	364-98-7	weak acid	24.7	-0.16
Diclofenac	15307-86-5	acidic	33.5	-0.67
Diprophylline	479-18-5	neutral	-4.0	-0.24
Ethinyl estradiol	57-63-6	neutral	46.8	0.39
Famotidine	76824-35-6	weak base	15.7	-0.06
Felbamate	25451-15-4	neutral	19.1	-0.64
Felodipine	72509-76-3	neutral	46.1	0.35
Fenoprofen	31879-05-7	acidic	17.2	-1.27
Finasteride	98319-26-7	neutral	38.9	0.30
Floxacillin	5250-39-5	acidic	23.4	-0.56
Flumazenil	78755-81-4	neutral	18.4	-0.04
Flurbiprofen	5104-49-4	acidic	26.8	-1.56
Furosemide	54-31-9	acidic	21.4	-0.71
Gemfibrozil	25812-30-0	acidic	32.9	-1.34
Glipizide	29094-61-9	acidic	21.1	-0.73
Griseofulvin	126-07-8	neutral	33.1	0.12
Hydrochlorothiazide	58-93-5	weak acid	15.9	-0.13
Hydrocortisone	50-23-7	neutral	27.9	0.12
Imipramine	50-49-7	basic	51.6	1.16
Indomethacin	53-86-1	acidic	25.3	-0.96
Isradipine	75695-93-1	neutral	40.0	0.13
Ketoconazole	65277-42-1	weak base	42.9	0.32
Ketoprofen	22071-15-4	acidic	21.9	-1.01
Labetalol	36894-69-6	amphoteric	43.9	0.71
Lignocaine	137-58-6	basic	32.6	0.40
Methylprednisolone	83-43-2	neutral	32.1	0.16
Metronidazole	443-48-1	weak base	-3.3	-0.25
Minoxidil	38304-91-5	weak base	19.0	-0.04
Nabumetone	42924-53-8	neutral	38.4	0.03
Nadolol	42200-33-9	basic	20.2	-0.33
Nicardipine	55985-32-5	weak base	45.9	0.47
Nifedipine	21829-25-4	neutral	29.0	-0.04
Nitrendipine	39562-70-4	neutral	40.5	0.16
Papaverine	58-74-2	weak base	34.4	0.00
Pentoxifylline	6493-05-6.	neutral	12.0	-0.10
Perphenazine	58-39-9	basic	56.3	1.35
Phenytoin	57-41-0	weak acid	31.6	0.03
Pindolol	13523-86-9	basic	42.0	0.85
Prazosin	19216-56-9	weak base	31.6	-0.05
Prednisolone	50-24-8	neutral	28.0	0.14
Prednisone	53-03-2	neutral	25.9	0.05
Primidone	125-33-7	neutral	8.9	-0.15
Probenecid	57-66-9	acidic	20.1	-0.75
Procainamide	614-39-1	basic	19.9	0.02
Propanolol	525-66-6	basic	50.8	1.29
Propylthiouracil	51-52-5	weak acid	3.9	-0.34
Proxyphylline	603-00-9	neutral	1.1	-0.85
Sulfachlorpyridazine	80-32-0	acidic	6.0	-0.76
Sulfameter	651-06-9	acidic	3.7	-0.61
Sulfamethoxypyridazine	80-35-3	weak acid	9.6	-0.59
Sulfinpyrazone	57-96-5	acidic	26.1	-0.65
Sulfisoxazole	127-69-5	acidic	2.9	-0.72
Sulphadimidine	57-68-1	amphoteric	34.6	0.22
Sulpiride	15676-16-1	basic	25.5	0.12
Tamoxifen	10540-29-1	basic	58.7	1.48
Theobromine	83-67-0	weak acid	-4.1	-0.25
Tolfenamic Acid	13710-19-5	acidic	36.6	-0.76
Trazodone	19794-93-5	weak base	36.3	0.01
Trimethoprim	738-70-5	weak base	20.8	-0.06
Warfarin	81-81-2	acidic	19.9	-0.78

**Table 3.** The CHI (IAM) obtained from the literature [13] (CHI (IAM) reference), the CHI (IAM) values obtained on the new IAM.PC.DD2 columns and the acid/base character of the IAM calibration compounds

Compound	Acid/base character	CHI (IAM)	CHI (IAM) “new”
Carbamazepine	neutral	28.9	27.0
Colchicine	neutral	23.7	23.0
Warfarin	acidic	19.9	24.0
Indomethacin	acidic	32.5	30.0
Nicardipine	weak base	45.9	45.0
Propranolol	basic	45.1	42.0
Imipramine	basic	54.1	45.0
Ketoprofen	strong acid	21.9	20.0
Chlorpromazine	basic	61.9	54.0
Haloperidol	basic	44.3	48
Budesonide	neutral	38.7	38

**Table 4.** The experimental CHI (IAM) values of the investigated compounds obtained on the new IAM.PC.DD2 columns and the CHI (IAM) obtained from the literature [13] (reference CHI (IAM))

Compound	CHI (IAM) from ref [13]	R20511-014-3 CHI (IAM)	P20511-014-3 CHI (IAM)	log *K* HSA from ref [13]	log *K* HSA
Acecainide	23.30	17.72	17.57	0.69	0.52
Acetanilide	10.70	11.55	11.50	0.62	0.43
Acetazolamide	1.66	0.99	0.74	1.47	1.44
Alclofenac	17.80	20.47	21.63	5.11	5.40
Amoxapine	56.50	45.41	44.74	2.15	2.70
Bamethan	18.60	18.53	18.03	0.49	0.42
Betamethasone	31.70	30.18	30.05	1.09	1.39
Carbamazepine	26.50	27.18	27.07	1.99	1.81
Chlorpromazine	61.90	51.54	50.94	1.18	1.27
Cinoxacin	1.60	5.31	6.67	0.79	0.91
Colchicine	23.70	23.45	23.18	0.69	0.24
Cytarabine	-15.00	-6.76	-6.99	1.59	1.87
Diazoxide	24.70	23.39	23.44	5.42	5.58
Diclofenac	33.50	35.18	35.85	0.63	0.24
Diprophylline	-4.00	-1.49	-1.98	4.1	4.24
Ethinyl Estradiol	46.80	45.30	45.23	0.46	0.50
Famotidine	15.70	16.82	16.08	1.39	3.43
Felbamate	19.07	39.16	39.21	3.46	4.15
Felodipine	46.10	44.37	44.45	5.91	6.11
Fenoprofen	17.20	27.17	28.26	1.58	2.32
Finasteride	38.90	37.15	37.17	3.62	3.55
Floxacillin	23.40	23.76	24.59	0.56	0.65
Flumazenil	18.40	18.27	18.27	7.81	8.50
Flurbiprofen	26.80	30.23	30.89	1.26	4.01
Furosemide	21.41	21.96	22.69	3.64	8.50
Gemfibrozil	32.90	33.70	34.53	3.32	4.05
Glipizide	21.10	22.82	23.22	1.49	1.92
Griseofulvin	33.10	31.06	30.98	0.77	0.80
Hydrochlorothiazide	15.90	13.04	13.08	0.92	1.05
Hydrocortisone	27.90	27.23	27.02	1.95	2.59
Imipramine	51.60	45.58	44.81	6.17	5.59
Indomethacin	25.30	32.49	33.16	2.86	3.36
Isradipine	40.00	39.11	39.16	2.9	3.30
Ketoconazole	42.90	39.50	39.26	4.8	5.40
Ketoprofen	21.90	22.93	23.89	1.28	1.80
Labetalol	43.90	38.29	37.48	0.69	0.52
Lignocaine	32.60	24.89	25.53	1.35	1.53
Methylprednisolone	32.10	30.91	30.77	0.76	0.28
Metronidazole	-3.30	-2.71	-3.24	0.66	0.72
Minoxidil	19.00	15.93	16.08	3.16	3.41
Nabumetone	38.40	35.90	35.96	0.49	2.14
Nadolol	20.20	19.89	19.23	2.93	3.55
Nicardipine	45.90	43.82	43.73	1.41	1.95
Nifedipine	29.00	28.11	28.16	3.13	3.37
Nitrendipine	40.50	39.06	39.19	2.35	2.70
Papaverine	34.40	31.22	31.15	0.63	0.39
Pentoxifylline	12.00	14.04	13.87	3.27	4.03
Perphenazine	56.30	49.57	48.97	1.6	2.02
Phenytoin	31.60	30.18	30.20	0.54	0.63
Pindolol	42.00	25.69	25.31	1.81	2.40
Prazosin	31.60	29.82	29.35	0.89	0.95
Prednisolone	28.00	27.31	27.20	0.8	1.08
Prednisone	25.90	25.25	25.13	0.62	0.42
Primidone	8.90	10.31	10.49	3.44	4.01
Probenecid	20.10	23.45	24.38	0.76	0.49
Procainamide	19.90	11.73	11.89	0.73	1.67
Propranolol	50.80	42.24	41.46	0.63	1.02
Propylthiouracil	3.90	6.56	6.52	2.67	3.21
Proxyphylline	1.10	10.05	10.89	1.97	3.02
Sulfachlorpyridazine	6.00	10.08	10.83	2.04	2.20
Sulfameter	3.70	6.92	7.09	4.09	2.44
Sulfamethoxypyridazine	9.60	11.11	11.16	2.33	4.27
Sulfinpyrazone	26.10	26.19	26.89	3.17	2.66
Sulfisoxazole	2.90	6.48	7.37	1.54	3.68
Sulphadimidine	34.55	10.89	11.09	0.75	1.72
Sulpiride	25.50	15.80	16.15	4.84	0.71
Tamoxifen	58.70	54.23	53.78	1.29	4.82
Theobromine	-4.10	-3.16	-3.71	4.68	0.27
Tolfenamic Acid	36.60	40.72	41.53	2.46	6.60
Trazodone	36.30	34.09	33.86	0.8	3.06
Trimethoprim	20.80	20.99	20.52	4.45	0.95
Warfarin	19.90	23.47	24.18	0.7	4.16

**Table 5.** The estimated log *V*_dss_ values of the investigated compounds using equation 4 and their acid/base character

Drug name	Acid/base character	R20511-014-3 log *V*_dss_	P20511-014-3 log *V*_dss_	reference log *V*_dss_
Acecainide	basic	-0.03	-0.03	0.1
Acetanilide	neutral	-0.12	-0.12	-0.13
Acetazolamide	neutral	-0.47	-0.47	-0.46
Alclofenac	acidic	-1.04	-1.02	-1.1
Amoxapine	basic	0.62	0.57	1.67
Bamethan	basic	0.01	0	0.01
Betamethasone	neutral	0.12	0.12	0.18
Carbamazepine	neutral	-0.07	-0.07	-0.09
Chlorpromazine	basic	0.88	0.82	2.15
Cinoxacin	acidic	-0.38	-0.37	-0.42
Colchicine	neutral	0.02	0.01	0.03
Cytarabine	weak base	-0.26	-0.26	-0.31
Diazoxide	weak acid	-0.19	-0.19	-0.16
Diclofenac	acidic	-0.6	-0.57	-0.67
Diprophylline	neutral	-0.22	-0.23	-0.24
Ethinyl estradiol	neutral	0.28	0.27	0.39
Famotidine	weak base	-0.04	-0.05	-0.06
Felbamate	neutral	0.07	0.07	-0.64
Felodipine	neutral	0.23	0.23	0.35
Fenoprofen	acidic	-1.02	-0.98	-1.27
Finasteride	neutral	0.21	0.21	0.3
Floxacillin	acidic	-0.55	-0.53	-0.56
Flumazenil	neutral	-0.04	-0.04	-0.04
Flurbiprofen	acidic	-1.44	-1.42	-1.56
Furosemide	acidic	-0.7	-0.68	-0.71
Gemfibrozil	acidic	-1.31	-1.27	-1.34
Glipizide	acidic	-0.69	-0.68	-0.73
Griseofulvin	neutral	0.04	0.03	0.12
Hydrochlorothiazide	weak acid	-0.18	-0.17	-0.13
Hydrocortisone	neutral	0.1	0.09	0.12
Imipramine	basic	0.66	0.6	1.16
Indomethacin	acidic	-0.72	-0.69	-0.96
Isradipine	neutral	0.08	0.08	0.13
Ketoconazole	weak base	0.12	0.1	0.32
Ketoprofen	acidic	-0.98	-0.96	-1.01
Labetalol	amphoteric	0.38	0.34	0.71
Lignocaine	basic	0.14	0.16	0.4
Methylprednisolone	neutral	0.12	0.11	0.16
Metronidazole	weak base	-0.24	-0.25	-0.25
Minoxidil	weak base	-0.11	-0.1	-0.04
Nabumetone	neutral	-0.09	-0.09	0.03
Nadolol	basic	-0.34	-0.35	-0.33
Nicardipine	weak base	0.32	0.32	0.47
Nifedipine	neutral	-0.07	-0.07	-0.04
Nitrendipine	neutral	0.08	0.08	0.16
Papaverine	weak base	-0.13	-0.13	0
Pentoxifylline	neutral	-0.07	-0.07	-0.1
Perphenazine	basic	0.66	0.61	1.35
Phenytoin	weak acid	-0.02	-0.02	0.03
Pindolol	basic	0.14	0.13	0.85
Prazosin	weak base	-0.11	-0.13	-0.05
Prednisolone	neutral	0.12	0.12	0.14
Prednisone	neutral	0.03	0.03	0.05
Primidone	neutral	-0.13	-0.13	-0.15
Probenecid	acidic	-0.66	-0.64	-0.75
Procainamide	basic	-0.13	-0.12	0.02
Propanolol	basic	0.64	0.59	1.29
Propylthiouracil	weak acid	-0.31	-0.31	-0.34
Proxyphylline	neutral	-0.75	-0.74	-0.85
Sulfachlorpyridazine	acidic	-0.71	-0.7	-0.76
Sulfameter	acidic	-0.57	-0.57	-0.61
Sulfamethoxypyridazine	weak acid	-0.57	-0.56	-0.59
Sulfinpyrazone	acidic	-0.64	-0.62	-0.65
Sulfisoxazole	acidic	-0.68	-0.67	-0.72
Sulphadimidine	amphoteric	-0.41	-0.41	0.22
Sulpiride	basic	-0.11	-0.1	0.12
Tamoxifen	basic	0.94	0.89	1.48
Theobromine	weak acid	-0.24	-0.25	-0.25
Tolfenamic Acid	acidic	-0.54	-0.49	-0.76
Trazodone	weak base	-0.09	-0.1	0.01
Trimethoprim	weak base	-0.05	-0.06	-0.06
Warfarin	acidic	-0.69	-0.68	-0.78
